# Endometrial Stem/Progenitor Cells–Their Role in Endometrial Repair and Regeneration

**DOI:** 10.3389/frph.2021.811537

**Published:** 2022-01-20

**Authors:** Fiona L. Cousins, Caitlin E. Filby, Caroline E. Gargett

**Affiliations:** ^1^The Ritchie Centre, Hudson Institute of Medical Research, Clayton, VIC, Australia; ^2^Department of Obstetrics and Gynecology, Monash University, Clayton, VIC, Australia

**Keywords:** stem/progenitor cells, endometrium, menstruation, regeneration, repair, endometriosis

## Abstract

The human endometrium is a remarkable tissue, undergoing ~450 cycles of proliferation, differentiation, shedding (menstruation), repair, and regeneration over a woman's reproductive lifespan. Post-menstrual repair is an extremely rapid and scar-free process, with re-epithelialization of the luminal epithelium completed within 48 h of initiation of shedding. Following menstruation, the functionalis grows from the residual basalis layer during the proliferative phase under the influence of rising circulating estrogen levels. The regenerative capacity of the endometrium is attributed to stem/progenitor cells which reside in both the epithelial and stromal cell compartments of the basalis layer. Finding a definitive marker for endometrial epithelial progenitors (eEPCs) has proven difficult. A number of different markers have been suggested as putative progenitor markers including, N-cadherin, SSEA-1, AXIN2, SOX-9 and ALDH1A1, some of which show functional stem cell activity in *in vitro* assays. Each marker has a unique location(s) in the glandular epithelium, which has led to the suggestion that a differentiation hierarchy exists, from the base of epithelial glands in the basalis to the luminal epithelium lining the functionalis, where epithelial cells express different combinations of markers as they differentiate and move up the gland into the functionalis away from the basalis niche. Perivascular endometrial mesenchymal stem cells (eMSCs) can be identified by co-expression of PDGFRβ and CD146 or by a single marker, SUSD2. This review will detail the known endometrial stem/progenitor markers; their identity, location and known interactions and hierarchy across the menstrual cycle, in particular post-menstrual repair and estrogen-driven regeneration, as well as their possible contributions to menstruation-related disorders such as endometriosis and regeneration-related disorder Asherman's syndrome. We will also highlight new techniques that allow for a greater understanding of stem/progenitor cells' role in repair and regeneration, including 3D organoids, 3D slice cultures and gene sequencing at the single cell level. Since mouse models are commonly used to study menstruation, repair and regeneration we will also detail the mouse stem/progenitor markers that have been investigated *in vivo*.

## Introduction

### The Menstrual Cycle

The human endometrium undergoes ~450 cycles of proliferation, differentiation, breakdown, shedding, and repair across a woman's reproductive lifespan. The endometrium is composed of two layers. The basalis is adjacent to the muscular myometrium and is not shed during menstruation, and it is from this layer that the upper layer of the endometrium, the functionalis, arises during each menstrual cycle (1). The functionalis undergoes the most structural changes throughout the menstrual cycle in response to ovarian-derived steroids 17β estradiol and progesterone (2).

The estradiol-dominant proliferative phase begins on approximately day 4 of an average cycle (3), stimulating proliferation of the glandular epithelium, the vasculature and stroma. Estradiol primes the endometrium for the structural changes that it will undergo during the secretory phase by inducing estrogen-dependent expression of the progesterone receptor (4). During the secretory phase, progesterone is secreted by the corpus luteum following ovulation. Epithelial cell proliferation decreases, stromal cells undergo cellular enlargement to become pre-decidual cells. During the mid-secretory phase decidualization of pre-decidual cells occurs under the luminal epithelium and around spiral arterioles. By the late secretory phase, the decidua is infiltrated by T cells, uterine natural killer cells and macrophages (5).

In the absence of an implanted blastocyst, the corpus luteum regresses resulting in a rapid decrease in ovarian-derived steroid production. Progesterone withdrawal initiates menstruation, a cascade of events that results in the piecemeal shedding (6) of the functionalis and expulsion of tissue via the vagina. Whilst outward bleeding may last for up to 5 days in some women, repair processes have been initiated from day 1. Scanning electron microscopy (SEM) studies show that re-epithelialization of the endometrium occurs within 48 h and in a piecemeal fashion (7). The endometrium is unique in that it displays unparalleled tissue remodeling following menstruation, resulting in a scar-free tissue (6). Furthermore, this process occurs in a steroid hormone-depleted environment, as evidenced in animal models of endometrial repair (8, 9).

Re-epithelialization of the endometrium is thought to occur by two mechanisms. The first was proposed by Novak and Te Linde in 1924, who suggested the new luminal epithelium arises from the residual basalis glands (10). SEM studies of menstrual phase endometrium show epithelial extensions attached to basal glands (7). The second mechanism is that of mesenchymal to epithelial transition (MET) where stromal cells in the basalis undergo cellular transformation to become new luminal epithelial cells. Three studies have reported low epithelial cell proliferation during post-menstrual repair, along with isolated epithelial cells on the surface of the endometrium, unassociated with the glandular epithelium (11–13). The role of MET has also been investigated in mouse models of menstruation/post-partum repair, which would indicate that the stroma does contribute in some part to the luminal epithelium (14–16) but it is likely the glandular epithelium is the main contributor to the new luminal surface.

Regeneration of the endometrium following repair is an estrogen-dependent process, whereby the endometrium grows from a post-menstrual depth of 0.5 to 7–8 mm during the mid-proliferative phase (17). This highly regenerative capacity is likely driven by stem/progenitor cell populations that reside in the basalis.

In this review we will focus on stem/progenitor populations that are likely involved in menstruation, repair, and early regeneration of the tissue as well as those populations which may contribute to menstrual/regeneration disorders such as endometriosis and Asherman's syndrome.

## Human Stem/Progenitor Cells in Menstruation, Endometrial Repair, and Regeneration

Adult stem cells are rare, undifferentiated cells found in most tissues and organs with the unique properties of self-renewal to maintain the stem cell pool and differentiation to generate the functional cells of the tissue in which they reside (18). Paradoxically, these stem/progenitor cells are often quiescent and rarely proliferate. Their transit amplifying daughter cells rapidly expand to ensure cellular replacement in regenerating tissues. It was initially hypothesized that endometrial stem/progenitor cells would be located in the basalis, as it remained during menstruation and provided a cellular source to regenerate the functionalis in the following cycle (19, 20). The epithelial cells of the basalis are quiescent and only proliferate occasionally, while functionalis glandular epithelium acts as the rapidly proliferating transit amplifying population in endometrial regeneration (20).

### Endometrial Epithelial Progenitors

**Figure 1:**
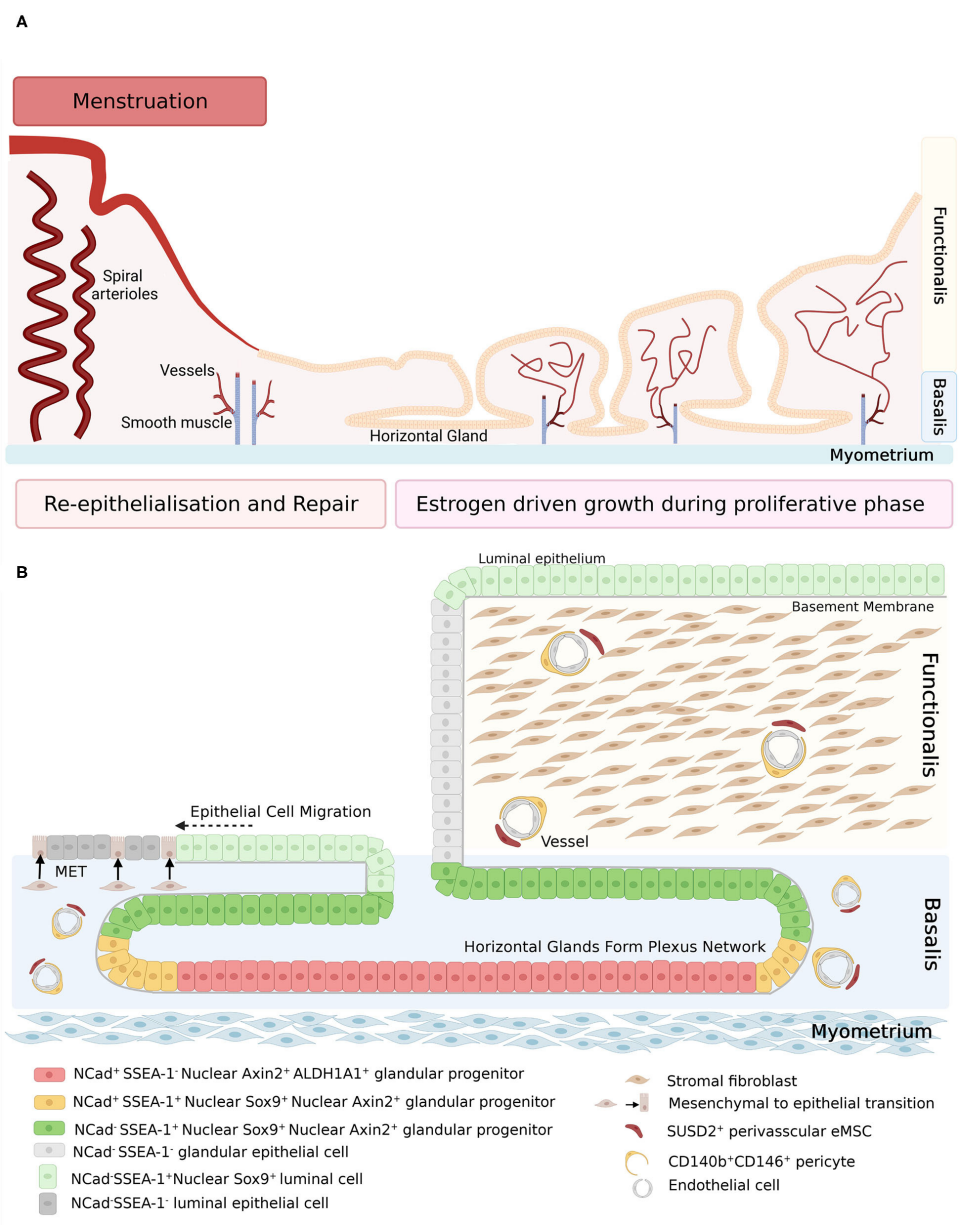
**(A)** Schematic of human endometrium in menstrual and proliferative phases. **(B)** Schematic of human endometrial mesenchymal stem cell and epithelial stem/progenitor hierarchy in menstruating and proliferating endometrium. During re-epithelization, SSEA-1^+^ epithelial cells (light green) migrate from the stumps of residual glands across the denuded surface, with some evidence of mesenchymal to epithelial transition of stromal fibroblasts (brown). During the proliferative phase, regeneration of the endometrium is initiated by clonogenic epithelial stem/progenitor cells that form a hierarchy in the basalis glands (red, yellow, and dark green cells), followed by rapid proliferation of functionalis epithelial cells as the vertical glands elongate. Endometrial mesenchymal stem cells are localized around blood vessels in the functionalis and basalis. Created with BioRender.com, adapted from (27).

Human endometrial epithelial progenitors were first identified as rare clonogenic cells comprising 0.22% of the epithelial cell adhesion molecule positive (EpCAM^+^) epithelial cell population from hysterectomy tissue which includes the basalis (21). Subsequently, the stem cell properties of self-renewal, high proliferative potential, and differentiation into large gland like structures in 3D cultures were demonstrated *in vitro* for individual large endometrial clonogenic epithelial cells (22). Specific markers of basalis epithelial cells were then identified; AXIN2 (23, 24), SSEA-1 and nuclear SOX9 (nSOX9) (25). The first specific surface marker enriching for clonogenic epithelial cells, N-cadherin encoded by *CDH2*, was identified using an unbiased gene profiling approach comparing EpCAM^+^ endometrial epithelial cells from pre- and post-menopausal women (26). A potential epithelial hierarchy was also identified, based on the location (niche) of the N-cadherin^+^ cells in the bases of the branching glands in the basalis adjacent to the myometrium. N-cadherin^+^ SSEA-1^+^ nSOX9^+^ epithelial cells were proximal to N-cadherin^+^SSEA-1^−^ cells and N-cadherin^−^ SSEA-1^+^ nSOX9^+^ (Figure 1) more proximal again to occupy an ill-defined functionalis-basalis junction. The majority of the functionalis comprised epithelial cells negative for N-cadherin, SSEA-1 and nSOX9. However, the luminal epithelium is SSEA-1^+^ nSOX9^+^, most likely due to rapid re-epithelializing of the raw surface by these cells migrating from the remaining gland stumps during menstruation (Figure 1), indicating their role in endometrial repair (27, 28). The ALDH1A1 isoform of ALDH co-localizes with 78% of N-cadherin^+^ epithelial cells by immunofluorescence and confocal microscopy (29), suggesting a role for retinoic acid signaling in the progenitor function of N-cadherin^+^ epithelial cells. EpCAM^+^ N-cadherin^+^, EpCAM^+^ SSEA-1^+^ and the very rare EpCAM^+^ N-cadherin^+^ SSEA-1^+^ epithelial cells have been detected and quantified in menstrual fluid (30, 31), indicating that small populations of these cells are shed during menstruation (see section Role of Stem/Progenitor Cells in Menstrual Disorders/Regeneration Disorders).

Atrophic post-menopausal endometrial epithelium also contains N-cadherin^+^ epithelial cells in the bases of atrophic glands adjacent to the myometrium (26). A similar endometrial epithelial hierarchy has been identified in post-menopausal women taking estrogen replacement therapy in a fully regenerated endometrium with a basalis and functionalis. Atrophic post-menopausal endometrial epithelium also contains nuclear AXIN2^+^ epithelial cells (23).

New information on endometrial cell lineages using next generation sequencing technologies at the single cell level is rapidly being generated. Since most studies have been undertaken on endometrial biopsies (32–34), gene expression signatures for basalis epithelial cells have not always been available. However, a Visium spatial transcriptomics study of several cadaveric full thickness uterine tissues has captured signatures of luminal, glandular, and basalis epithelium and revealed that *SOX9*-expressing epithelial cells with a cell-cycling profile are widely distributed in proliferative-stage endometrium (34). Although, this Visium spatial analysis was unable to determine if *SOX9* was expressed in the nucleus or cytoplasm, it is possible that nSOX9^+^SSEA-1^+^ epithelial cells may extend further into the functionalis than first observed and behave as transit amplifying cells that contribute to the rapidly expanding glandular epithelium during endometrial regeneration. However, the *SOX*9^−^ expressing basalis epithelial cells have a non-cycling gene expression profile indicating their quiescence, as shown many years ago in tritiated thymidine incorporation *ex vivo* into endometrial tissue (2). Mouse endometrial epithelial progenitor cells were first identified as quiescent label retaining cells (LRC) by pulse-chase experiments using bromo-deoxyuridine (BrdU), a DNA synthesis label, to detect rarely dividing cells which retain the label (35, 36). Mouse endometrial epithelial LRC were identified in the luminal epithelium and did not express nuclear ERα (35), but were the first cells to proliferate on estrogen replacement of ovariectomised BrdU-labeled LRC mice, thereby driving endometrial luminal and glandular regeneration (37).

More recently, a single cell pulse-chase lineage tracing study using *Cre-loxP-Keratin19* reporter system was used to identify mouse endometrial epithelial stem cells and their niche (38). The epithelial stem cells which generated EpCAM^+^ FoxA2^−^ luminal epithelial cells and EpCAM^+^ FoxA2^+^ glandular epithelial cells were located in the intersection zone of the luminal and glandular epithelium. They had capacity to repair the luminal epithelium and regenerate the glandular component over numerous estrus cycles and following pregnancy. Other lineage tracing studies have identified *Lgr5*^+^-expressing cells at the tips of the glands invaginating into the uterine mesenchyme in neonatal mice (39) and *Axin2*-expressing epithelial cells deep in the gland bases of adult mice which regenerated endometrial glands during estrus cycling (24). It appears that there are several stem/progenitor populations in mouse endometrium that are responsible for endometrial repair and regeneration, however the hierarchy of these stem/progenitor cells is yet to be determined.

### Endometrial Mesenchymal Stem Cells

Most postnatal tissues, whether highly regenerative or not, contain a population of mesenchymal stem cells (MSC), including human endometrium (eMSC) (40). MSC were first identified in bone marrow aspirates as clonogenic cells with a fibroblastic morphology (CFU-F) with capacity to differentiate into multiple mesodermal lineages (41). MSC were later defined by the International Society for Cell & Gene Therapy (ISCT) as plastic adherent stromal cells that differentiated into adipocytes, chondrocytes and osteocytes *in vitro* and had a characteristic surface marker phenotype distinguishing them from haemopoietic stem cells (42). However, stromal fibroblasts also fulfill the ISCT criteria, which does not distinguish clonogenic MSC with adult stem cell properties (self-renewal, proliferative potential and differentiation *in vivo*) (43). More recently, functional and morphological studies have identified numerous tissues with perivascular MSC with adult stem cell function (44, 45), including human endometrium (46, 47). In this review we will focus on perivascular endometrial MSC (eMSC) rather than endometrial stromal fibroblasts. For a more detailed discussion on the differences between perivascular eMSC and endometrial stromal fibroblasts in endometrial tissue and menstrual fluid see Bozorgmehr et al., (48).

The endometrium has a substantial vascularized stroma which regenerates during the proliferative stage of each menstrual cycle, and is likely mediated by eMSC. eMSC were first identified as clonogenic stromal cells (1.25% of stromal cells) (21, 49), which fulfill the ISCT criteria and also undergo self-renewal and *in vitro* differentiation to multiple mesodermal lineages (22). Their perivascular niche was discovered when specific surface markers were identified that enriched for the clonogenic endometrial stromal cells, first as pericytes co-expressing CD140b (PDGFRβ) and CD146 (46) and as SUSD2^+^ perivascular cells (47).

SUSD2^+^ eMSC can also generate endometrial stromal tissue *in vivo* when transplanted underneath the kidney capsule (47). In this model, SUSD2^+^ eMSC generate vimentin^+^ fibroblasts and induce the migration of endothelial cells that promote angiogenesis (47). The pro-angiogenic activity of eMSC has also been shown *in vitro* (50). Taken together, these data suggest that eMSC may be responsible for stromal vascular regeneration of the endometrium during the proliferative phase, mediated by both growth and differentiation and also by paracrine effects promoting angiogenesis.

These perivascular eMSC were identified around small and large blood vessels in both the basalis as expected and also in the functionalis, which indicated they would be shed in menstrual fluid (see section Role of Stem/Progenitor Cells in Menstrual Disorders/Regeneration Disorders). Gene expression profiling has demonstrated that CD140b^+^CD146^+^ eMSC rapidly lose their gene signature in culture and adopt the CD140b^+^CD146^−^ stromal fibroblast signature (51). This suggests eMSC differentiate into stromal fibroblasts, further contributing to the confusion between MSC and stromal fibroblasts (52). However, this differentiation has not been identified *in vivo*. SUSD2^+^ and SUSD2^−^ endometrial cells differentiate into decidual cells with similar but distinct gene profiles and both contribute to the formation of the maternal placenta (53–55).

Other markers of perivascular eMSC include NG2, Stro-1, EphA3, W8B2, and CD271 and CD34 which are found in the adventitia of blood vessels rather than a pericyte or medial location [reviewed in (48, 56)]. However, the CD34 population failed to regenerate human endometrium in functional studies (57). The perivascular niche suggests that eMSC likely contribute to vascular and stromal regeneration each menstrual cycle.

Endometrial thickness can be restored in post-menopausal women via oral estrogen therapy, suggesting that endometrial stem/progenitor populations remain quiescent after menopause but when exposed to exogenous estrogen rapidly respond to regenerate both glands and stromal vascular tissue (26). Like N-cadherin^+^ eEPC, SUDS2^+^ eMSC can be isolated from post-menopausal (PM) endometrium (58). They have a lower cloning efficiency than in pre-menopausal endometrium, but are detected in similar numbers (58).

Single cell RNA sequencing of human endometrium has identified a small smooth muscle population expressing *SUSD2*, CD146 (*MCAM*), and CD140b (*PDGFRB*) (32). In mouse endometrium, scRNAseq of *Pdgfrb*-BAC-eGFP reporter mice also identified a perivascular population of *Pdgfrb*^+^ *MCAM*^+^ cells with a perivascular gene profile and perivascular niche *in vivo*, that was distinct from 3 novel endometrial fibroblast populations also identified (59). These studies confirm our biological findings and provide further insight into the role of perivascular eMSC in endometrial regeneration.

### Bone Marrow Derived Stem Cells

Bone marrow derived stem cells (BMDSC) have been suggested as an exogenous source of stem cells in the endometrium (60). In humans, it has been reported that BMDSC contribute up to 48% of the epithelium and 52% of the stroma (60). Most of the supporting data has been generated from mouse models, which have shown BMDSC contribute to epithelial, stromal, and endothelial lineages (61–64). However, a 2018 study disputed their contribution. Using two different transgenic fluorescent tagged mouse lines to repopulate the bone marrow of irradiated recipient mice and sophisticated imaging and microscopy, Ong et al. demonstrated that BMDSC did not contribute to epithelial or stromal lineages (65). Instead they highlighted that intra-epithelial and stromal-derived cells were CD45^+^ leukocytes and that bone marrow-derived macrophages failed to immunostain with CD45 (65). This highlighted the limitations in the previous body of work relying solely on the identification of CD45^+^ cells. BMDSC contribution to other body organs has been similarly controversial but the body of evidence suggests their plasticity or ability to transdifferentiate does not occur for similar technical issues (66). For the purpose of this review we will be focusing on endometrial-derived eMSC and eEPC.

### Menstrual Fluid

Menstrual fluid, discharged via the vagina following declining circulating progesterone levels, is a complex fluid containing shed endometrial tissue (31, 48), secreted proteins (67, 68), immune cells, peripheral blood components, vaginal epithelial cells, clots, and mucous (69). Shed menstrual fluid cells mainly comprise CD45^+^ leukocytes (>90%), with the remainder being endometrial cells (31, 69). Menstrual fluid endometrial cells include stromal cells, for example fibroblasts (69), SUSD2^+^ eMSC (70), and epithelial cells such as N-cadherin^+^ and SSEA-1^+^ eEPC (31). A similar proportion of these stem/progenitor cells is identified in menstrual fluid compared to endometrium (31, 70). Both SUSD2^+^ eMSC and N-cadherin^+^ eEPC have also been detected and their abundance reported in menstrual blood collected from the uterine cavity during surgery before efflux into the vagina (30). The proportion of clonogenic units in menstrual fluid endometrial cells is comparable for epithelial clones (0.31% for menstrual fluid and 0.22% for eutopic tissue) and somewhat lower for stromal clones (0.22% for menstrual fluid and 1.25% for eutopic tissue) than in endometrial tissue (31).

Menstrual fluid also contains cells described broadly as menstrual stem cells (MenSC) that fulfill the ISCT criteria which are easily isolated based on their adherence to plastic. However, they are likely heterogeneous due to non-specific and non-standardized isolation procedures (48, 71, 72). MenSC express markers for MSC, with the exception of STRO-1 (70, 73). MenSC likely comprise a combination of eMSC and predominantly endometrial stromal fibroblasts. A comparison of MenSC and eMSC has been extensively reviewed elsewhere, which highlights the need for standard isolation and characterization of MenSC (48).

Viable eMSC can be reliably isolated from menstrual fluid, their proportions studied, their clonogenicity assessed (31), and their behavior characterized *in vitro* (70). Together this indicates that menstrual eMSC are a reliable source for research that characterizes the biology of eMSC. Menstrual eMSC do tend to be more apoptotic and necrotic than other sources of MSC, however they can be prevented from undergoing apoptosis and senescence by A8301 treatment (70), thus indicating menstrual eMSC are also a viable option for cellular therapy (48).

## Stem/Progenitors in *in vitro* Models of Menstruation, Repair and Regeneration

### 3D Endometrial Organoids/Menstrual Fluid Organoids

**Figure 2:**
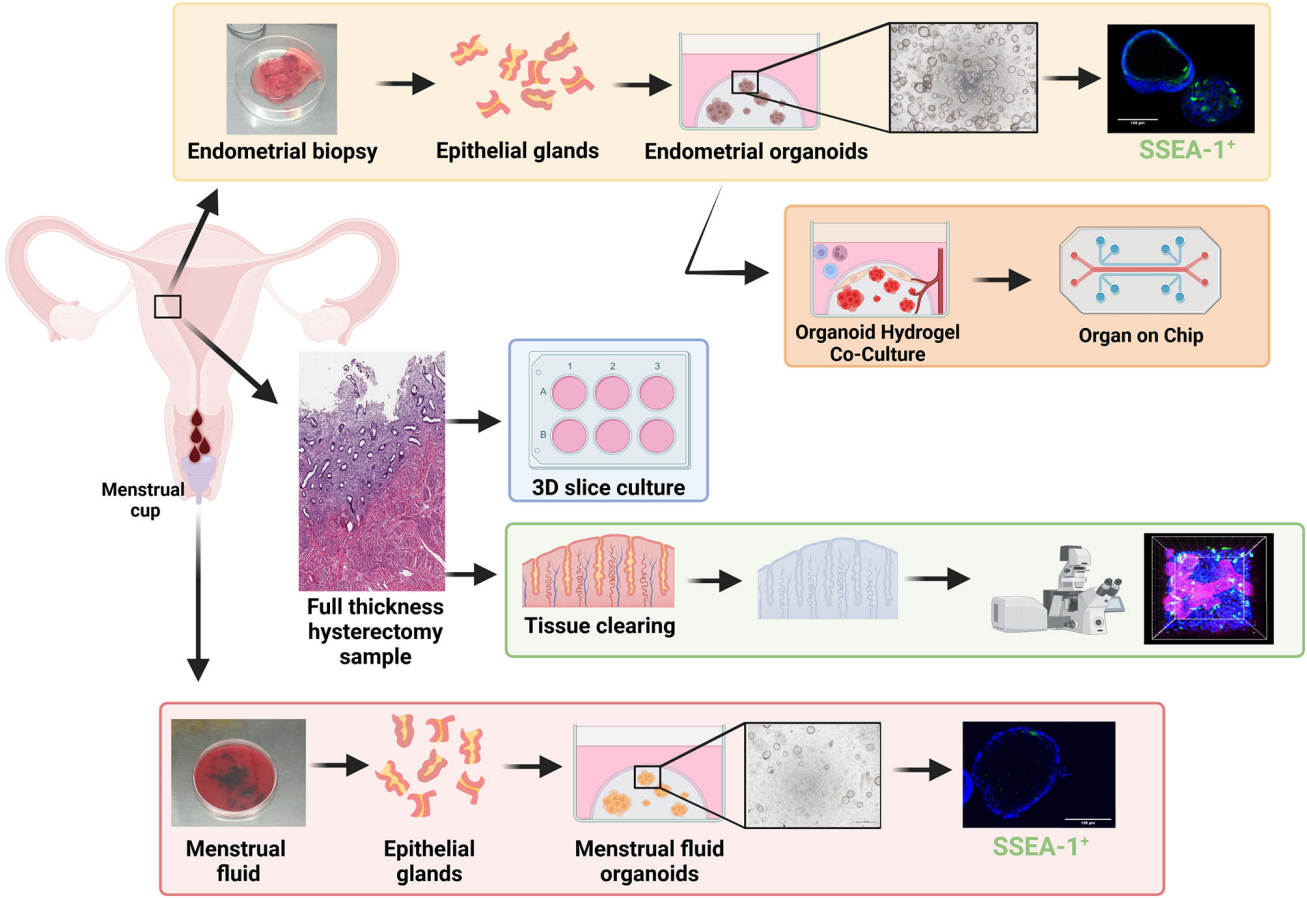
Stem/progenitor cells in *in vitro* models of repair and regeneration. Endometrial biopsies can be used in 3D organoid experiments including single cell organoid culture (yellow box) and hydrogel-based co-culture and organ-on-chip systems (orange box) which show great promise for future endometrial research. Full thickness endometrium collected at hysterectomy has been used for; tissue clearing (green box) to visualize structures in 3D via high powered microscopy and used for 3D slice cultures (blue box). Menstrual fluid can be easily collected in a menstrual cup, from which menstrual fluid organoids (red box) can be generated. Created with BioRender.com.

The study of human primary endometrial epithelial biology has previously been limited to 2D culture such as (1) epithelial monolayers, which either become overgrown by stromal cells or senesce (74, 75), (2) colony forming unit assays, seeded at very low density and generally terminal experiments that do not permit long-term culture or functional assays (21, 49), or (3) serial subcloning which permits up to four passages for large colonies derived from CFU-F (22). However, our recent evidence that the endometrium contains N-cadherin^+^ and putative SSEA-1^+^ eEPC (26, 76) capable of forming gland-like structures in 3D culture maintained in ECM, together with organoid technology (77, 78), has enabled generation of human endometrial organoids (EMO) using defined serum-free culture conditions (79, 80) (Figure 2, yellow box).

EMO have since been derived from biopsies, hysterectomy tissue, endometrial cancer, endometrial hyperplasia, endometriosis lesions from various locations, and placental decidual tissue (79–81). They can be expanded for >6 months in culture, cryopreserved and generated from cryopreserved gland fragments (82) and show responsiveness to estrogen and progesterone (79, 83). The gene expression profile of EMO has been studied at both the bulk and single cell level (79, 80, 83, 84), however many of these studies are limited by their use of endometrial biopsies instead of hysterectomy tissue, which contain the full hierarchy of eEPC, including those located in the rhizome-like glandular structures of the basalis (85, 86). Changes in EMO cell fate in response to hormones (83) and inhibition of key developmental signaling molecules (NOTCH) (87) enhance our understanding of endometrial epithelial cell fate trajectory and its role in development and disease—an area that has previously been very challenging to study. EMO also show promise for drug screening, demonstrating sensitivity to specific compounds (88).

EMOs are derived from bulk endometrial epithelial fragments and therefore comprise a heterogenous population. They potentially include contamination of surrounding stroma, which recede from culture after multiple passages, but may influence organoid formation and contribute to variation in patient-derived organoid lines. While the ability to generate single cell EMO (scEMO) from existing EMO cultures has been demonstrated (79, 80, 84), the ability of naïve single endometrial cells to form EMO has been underexplored. Generating scEMO from FACS-sorted epithelial subpopulations has potential for investigating the roles of epithelial progenitor cells in endometrial regeneration.

Organoids can be generated from shed endometrial tissue in menstrual fluid (MFO) (89, 90) (Figure 2, red box).While MFO are less abundant than EMO, they appear to represent EMO in proliferation rates, responses to hormones, and gene expression profiles (89, 90). They can also be derived from disease states including endometriosis and adenomyosis. They can also be derived from girls and young women without the need for an endometrial biopsy, enabling the study of early disease mechanisms, precision medicine, and diagnosis (91).

While EMO and MFO represent major advancements in studying menstrual biology, these systems largely support epithelial cultures in isolation, and lack the stromal, mesenchymal, vasculature, and immune cells important for a functioning endometrium. Biomaterial engineering has generated synthetic hydrogels (92, 93) that permit co-culture of endometrial epithelial and stromal cells and these are being rapidly applied to EMO (Figure 2, orange box) (81, 94, 95). They are also being applied to the cells/tissues required to support the endometrium such as the vasculature (92) and immune cells [reviewed in (96)]. Coculture in defined hydrogel systems has enabled development of organ-on-chip systems for disease modeling and low-cost drug screening for other organs and disease states (97)—their potential for endometrium, endometriosis, and adenomyosis are exciting prospects to be explored (96). Organ-on-chip and micro-physiological systems (Figure 2, orange box) have multiple advantages, including infinite tunability (cell input, matrix composition, hormonal, and nutrient delivery), scalability, enabling coupling for modeling the complexity of endometrial regeneration.

### *In vitro* 3D Slice Cultures

Whilst single cell and co-cultured 3D organoids overcome some of the issues of using 2D *in vitro* models to study endometrial dynamics, endometrial repair/regeneration is a multicellular/multizonal, tightly controlled process which has previously been difficult to replicate in a dish. 3D thin tissue slice cultures provide a culture system that maintains endometrial structure (98). These tissue slice approaches have shown that the tissues can respond to estrogen and progesterone over 21 days *in vitro* (Figure 2, blue box). Histology of the slices indicates that zone-specific changes *in vitro* mimic *in vivo* hormone responses (98). Whilst stem/progenitor populations were not assessed in this study, the authors did show non-specific transduction of lacZ via adenovirus-mediated gene delivery and therefore the model has promise for studying stem/progenitor populations and interactions in an “*in vivo*-like” system, however 3D endometrial slice cultures are limited to terminal experiments.

### Tissue Clearing

Historically, the location and expression profiles of endometrial stem/progenitor cells have been presented in 2D via standard immunohistochemistry/immunofluorescence of thin tissue sections. The introduction of tissue clearing, whereby a tissue sample is rendered optically transparent via solvent- or aqueous-based solutions, has enabled a more detailed morphological examination of the endometrium in 3D. Samples are fixed, permeabilized, and then cleared so that light scattering and absorption caused by cellular contents are minimized. Optimal tissue clearing maintains native tissue architecture and preserves fluorescent proteins (99) (Figure 2, green box). Two recent studies have reconstructed the 3D morphology of full thickness endometrium and have shown that basalis glands form a rhizozome-like plexus structure horizontally across the endometrial basalis using tissue clearing (85) or genetic lineage tracing (86), challenging the long held view of vertical glands extending from blind-ended glands in the basalis. This plexus structure remains during menstruation, from which branched glands arise and vertically penetrate the functionalis. 2D studies clearly indicate that a hierarchy of epithelial/stem progenitor cell types with specific markers exists extending from the basalis to the luminal epithelium. Now these new technologies are available it will be exciting to see whether this epithelial hierarchy can be reconstructed in 3D, whilst also investigating the relationship between MSCs and the endometrial vasculature across all cycle phases.

## Stem/Progenitors in Mouse Models of Repair and Regeneration

Like women, the mouse endometrium also responds to cyclical changes of ovarian-derived steroids. In the mouse this occurs over a much shorter timeframe, ~4 days. Pro-estrous and estrus mimic the proliferative phase of the human cycle, where increasing concentrations of estrogens result in ovulation, followed by a progesterone dominant metestrus (100). Unlike women, the mouse endometrium does not undergo spontaneous decidualization in the presence of progesterone, but requires an implanted blastocyst. In the absence of implantation, the endometrium is reabsorbed during diestrus, and the cycle begins again. Murine stem/progenitor markers involved in the cyclical turnover of the endometrium during the estrous cycle have been extensively studied, readers interested in this area are referred to our recent reviews (28, 48).

Despite their lack of menstruation, mice are routinely used as an *in vivo* model for menstruation, repair, and regeneration, using several different approaches. The most commonly used mouse model of menstruation (MMoM) was first described by Finn and Pope, where exogenous hormones were administered to ovariectomised mice to mimic a human cycle. Decidualization was artificially induced by a physical stimulus (sesame oil) to the endometrium, to mimic blastocyst implantation. Exogenous hormones were then withdrawn to stimulate a menses-like event (101). This model has been optimized to use silastic hormone-secreting pellets and transvaginal delivery of oil to reduce variation in the decidual response (14, 102). This model has been used to assess a role for stem/progenitor cells during repair (0–24 h after P4 withdrawal) and regeneration (24–72 h) of the endometrium at menses (14, 103). Pseudopregnancy models of menstruation (PMoM) are also used, where female mice are mated with a vasectomized male to induce decidualization and then ovariectomy or mifepristone to induce menstruation (16, 104). Both models exhibit overt bleeding, breakdown/shedding, re-epithelialization, and regeneration of the endometrium, however breakdown and initial repair occur over a shorter time period in the MMoM (24 h) compared to the PMoM (48–72 h).

An early study used BrdU pulse-chase to assess LRCs in the glandular and luminal epithelial compartments during re-epithelialization in the MMoM. Glandular epithelial cells (GE) retain BrdU for longer periods than luminal epithelial cells (LE) and GE strongly express ERα during initial repair of the endometrium (81.6% ERα positive) (103). Proliferation of LE significantly increases during re-epithelialization (repair phase), in contrast to GE which only commences proliferation once breakdown and repair are complete. These data suggest a stem/progenitor population in the residual basal glands that support the formation of glandular growth during the subsequent regenerative phase (103).

Mouse telomerase reverse transcriptase (mTert), a putative stem/progenitor marker in the regenerative intestine (105), marks rare stromal, epithelial, and leukocyte populations in the cycling mouse endometrium (106). They are positively regulated by estrogen (106) and negatively regulated by progesterone as shown by lack of mTert^+^ cells in the LE or GE prior to progesterone withdrawal in the MMoM (107). During repair, mTert^+^EpCAM^+^ cells are rare (0.08% of total EpCAM population) and localized to the repairing LE, and no mTert^+^ cells were identified in the GE. In the repairing LE, mTert^+^Ki67^−^ cells were localized next to mTert^−^Ki67^+^ clusters (107). This suggests that mTert^+^ cells are progenitor cells that are located in the residual LE and undergo asymmetrical division to form transit amplifying cells which contribute to form the new LE during the steroid-depleted window of epithelial repair. It is likely the GE-derived mTert^+^ cells are present, but estrogen supplementation has not been studied in this model. Interestingly, mTert does not co-localize with BrdU^+^ LRC or CD44 suggesting that mTert^+^ cells may identify different progenitor cell types within the LE (106).

The contribution of the stromal cell compartment to repair via mesenchymal-epithelial transition (MET) has also been studied using cytokeratin as an epithelial marker in combination with different mesenchymal markers. Using the MMoM double immunostaining for cytokeratin and vimentin during early and late repair revealed rare cells undergoing MET close to the repairing epithelium (Figure 1) (14). Cytokeratin^+^vimentin^+^ cells have also been observed in the stromal cell compartment of repairing endometrium in a PMoM (16), and Amhr2^+^cytokeratin^+^ cells were observed in the new luminal epithelium in a post-partum model of endometrial repair (15). A recent study using a number of lineage tracing mouse reporter lines disputed the role of MET in the endometrium, however all of those studies were either in intact estrous cycling mice or mice treated with tamoxifen to induce epithelial expansion and did not investigate endometrial repair or regeneration following a major remodeling event (menses-like or post-partum models) (108). Taken together these data suggest that during normal cyclical turnover each cell compartment supports its own cell type but when the endometrium needs to repair following a major shedding event, such as parturition, the stromal compartment supports re-epithelialization.

A more recent study has identified SM22α-derived CD34^+^KLF4^+^ cells as a putative stromal progenitor cell involved in endometrial regeneration (109). SM22α^+^CD34^+^ cells were located in the endometrial stroma below the repairing epithelium, where they also co-stained with the epithelial marker E-cadherin. Like other stem/progenitors, proliferation of SM22α^+^CD34^+^KLF4^+^ cells is likely mediated by estrogens. Deletion of SENP1 (SUMOendopeptidase-1) induced SUMOylation, which in turn promoted ERα expression in the repairing endometrium. SM22α^+^CD34^+^KLF4^+^ cell proliferation was significantly increased in SENP1sm22αKO mice in comparison to WT mice and an increase in transdifferentiation of stromal cells into epithelial cells was observed. Repair of the endometrium was completed by 72 h post-progesterone withdrawal in SENP1smKO mice compared to 96 h in WT mice (109). In addition, SENP1 likely mediates stem/progenitor regulation of regeneration, as deletion of SENP1 leads to epithelial hyperplasia (109), highlighting the importance of a tightly controlled repair and regeneration process to prevent endometrial dysfunction.

We acknowledge the limitation that mice do not naturally menstruate and therefore any information gleaned from mouse studies into stem/progenitor dynamics must be carefully related to human studies. At the time of writing, Axin2 appears to be the only marker that has a similar role in glandular epithelial cell turnover in both mice and humans.

## Role of Stem/Progenitor Cells in Menstrual Disorders/Regeneration Disorders

### Endometriosis

#### Retrograde Menstruation Theory

**Figure 3:**
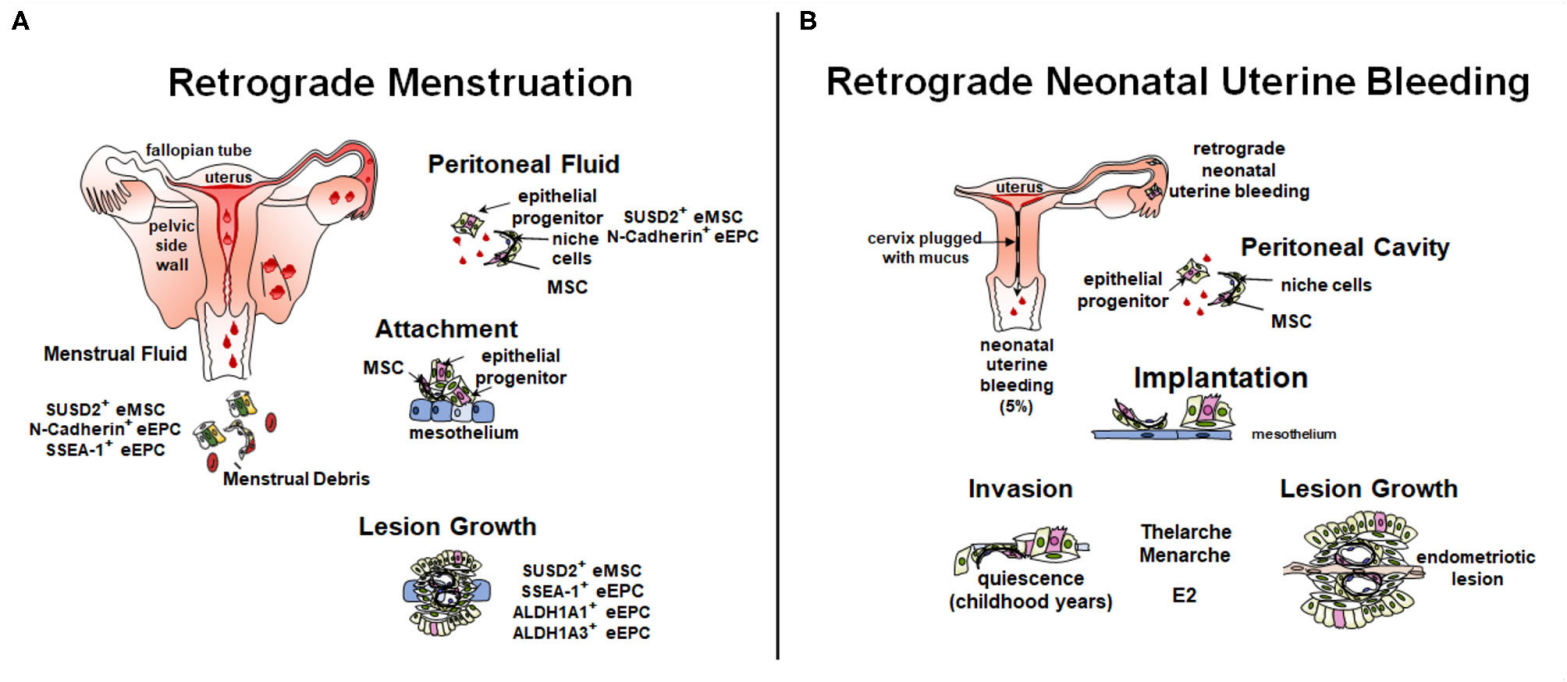
Role of stem/progenitor cells in the pathology of endometriosis. **(A)** In the adult, retrograde menstruation, results in stem/progenitor cells entering the peritoneal cavity. In some circumstances, stem/progenitor cells are able to survive and contribute to lesions establishment and progression. **(B)** Retrograde neonatal bleeding is observed in 5% of female born babies. Epithelial progenitors and eMSC enter the peritoneal cavity and remain quiescent until puberty, where increasing concentrations of estradiol (E2) stimulate lesion growth. Created with BioRender.com, adapted from (110).

Retrograde menstruation, where shedding endometrial fragments flow backwards through the Fallopian tubes and into the peritoneal cavity, is likely the main cause of endometriosis pathogenesis (Figure 3A). However, not all women who exhibit retrograde menstruation develop endometriosis. Other contributing factors likely play a part in determining who does and who does not develop endometriosis. The total number of endometrial cells is unlikely to play a role, given their similar prevalence in the peritoneal cavity of both women with and without endometriosis (30, 111). Rather, the type of cells deposited in the cavity may play a role in pathogenesis. Leyendecker et al. have suggested that endometriosis is caused by the shedding of basalis endometrium, as women with endometriosis have a higher prevalence of basalis fragments in their menstrual blood compared to controls (112). Furthermore, we have shown increased proportions of SSEA-1^+^ basalis cells in the functionalis layer normally shed with menstruation (76).

The initiation of lesions in the peritoneal cavity likely depends on the ability of stem/progenitor cells to adhere to, and for deep infiltrating endometriosis (DIE) invade, ectopic sites and subsequently give rise to epithelial and stromal progeny. Different theories exist on how they contribute to lesions (91, 110, 113). Factors likely contributing include the type of cells shed at menstruation, the influence of genetics such as, endometriosis risk variants and somatic mutations (114–117), site of attachment in the peritoneal cavity and the surrounding micro-environment. Stromal and epithelial stem/progenitor cells have been identified in menstrual blood, peritoneal fluid, and ectopic lesions (Figure 3A) (30, 31, 70, 118, 119).

#### Menstrual and Peritoneal Fluid Stem/Progenitor Cell Populations in Endometriosis

Study of endometrial stem/progenitor cells in menstrual and peritoneal fluid is still in its infancy, partly due to recent discovery of appropriate markers (26, 47, 76) and challenges in acquiring, processing, and analyzing complex fluids. Whilst easily acquired tissue fragments and stromal fractions have been studied by some, far fewer have attempted to identify, isolate, and characterize the endometrial stem/progenitor cells from menstrual fluid (48, 111). We identified clonogenic endometrial cells, SUSD2^+^ eMSC and N-cadherin^+^ eEPC concurrently in menstrual fluid and peritoneal fluid of women with and without endometriosis (31) and also clonogenic endometrial cells and SSEA-1^+^ eEPC in menstrual fluid of normal women (30, 31).

In menstrual fluid the proportions of SUSD2^+^ eMSC and SSEA-1^+^ eEPC endometrial stem/progenitor cells show minimal variation from one menstrual cycle to the next in both groups (31). On the other hand, the proportion of N-cadherin^+^ eEPC showed a poor agreement from one menstrual cycle to the next, indicating variability in the numbers of N-cadherin^+^ eEPC shed, likely due to their deep basalis location on the rhizomal-like gland structures. The concentration of SUSD2^+^ eMSC and N-cadherin^+^ eEPC in uterine menstrual blood appears similar between women with and without endometriosis (30).

Recently we have described the first ever stem/progenitor cell evidence of Sampson's 100 year-old theory of retrograde menstruation (30, 120). While we hypothesized that the concentrations of endometrial stem/progenitor cells retrogradely shed into the pelvic cavity would be higher in women with endometriosis, surprisingly our study did not find a significant difference in the concentrations of eMSC, eEPC, or clonogenic cells in peritoneal fluid during the menstrual phase of the cycle. This unexpected finding may be limited by sample size and a control group confounded by pelvic pain—women undergoing laparoscopy are not “normal” and thus a true control for peritoneal fluid is a rare occurrence (e.g., tubal ligations).

The clonogenic cells persisted in peritoneal fluid beyond the menstrual phase in women with endometriosis, whereas in controls they declined during the non-menstrual phase (30). This may indicate enhanced survival or persistent shedding of clonogenic cells in women with endometriosis. Other studies have shown an increased pro-invasive cytokine profile of peritoneal fluid from women with endometriosis (121) and this may aid the survival of stem/progenitor cells beyond the menstrual phase. Alternatively, the shed cells may exhibit different behavior due to underlying genetic or other regulatory programs (91). Finally, there was noticeable variation in the concentration of eMSC and N-cadherin^+^ eEPC in women with endometriosis, indicating the possibility of sub-groups with different pathophysiology that is worthy of further investigation.

#### Stem/Progenitors in Ectopic Lesions

Sequencing of epithelial and stromal cells in superficial and deep infiltrating lesions reveals characteristic somatic mutation profiles for each cell type derived from different clones suggesting each cell type is supported by their own stem/progenitor population (91, 115, 122). In support of this, both mesenchymal and epithelial stem/progenitor markers have been identified in ectopic lesions (25, 29, 29, 76, 123–125) (Figure 3).

The clonogenicity of eutopic and ectopic stromal and epithelial cells was lower for both epithelial and stromal cells from endometriomas when compared to matched eutopic endometrium (118). Lower cloning efficiency was also observed when control endometrium was compared to endometriomas. However, no significant difference was found in either cell population when eutopic endometrium from women with and without endometriosis was compared (118). That study did not compare the clonogenicity of either DIE or superficial endometriosis lesions, however a lower clonogenicity of endometrioma cells is in keeping with a lower organoid yield from ectopic rather than eutopic tissue (80). Furthermore, eMSC in ectopic lesions have a higher proliferative potential (119). Cultured endometrial stromal cells, fulfilling the ISCT criteria, show increased migration capacity, enhanced angiogenic potential, and exhibit altered expression of adhesion molecules in comparison to eutopic MSC (126). This suggests that the peritoneal cavity provides a micro-environment which promotes or selects for stem/progenitor cell activity. Perivascular SUSD2^+^ NTPDase2^+^ MSC have been identified in ovarian endometriomas via immunofluorescence (123).

Basalis epithelial stem/progenitor markers SSEA-1 and SOX9 are increased in eutopic secretory phase functionalis of endometriosis women in comparison to healthy controls (76). *In vitro*, these cells can form 3D gland-like structures highlighting their potential at supporting lesion development *in vivo*. SSEA-1^+^ cells are present in endometriosis lesions (25) as are the deep basalis epithelial markers ALDH1A1 and ALDH1A3 (29) and N cadherin (29, 124, 125), supporting Leyendecker's basalis theory of endometriosis pathogenesis.

#### Neonatal Uterine Bleeding

A new theory to explain the pathogenesis of pre-menarchial early onset endometriosis involves neonatal uterine bleeding (Figure 3B), a forgotten phenomenon occurring in ~5% of neonatal girls (127). Its incidence is highest in post-term babies. This neonatal vaginal bleed observed in the first week of life results from maternal progesterone withdrawal from the neonatal circulation upon birth. Unlike the mouse, the fetal uterus is fully formed *in utero* and autopsy studies have shown that the endometrium can undergo decidualization and there is evidence of endometrial shedding. The neonatal uterus is predominantly cervix which is functionally blocked with mucous thereby allowing any shedding endometrium to flow back into the pelvic cavity undetected, and only allowing minimal blood and cell numbers to permeate the cervical mucous. The neonatal endometrial stem/progenitor cells present in such shed endometrial tissue could invade the mesothelium and remain dormant in a similar manner to the dormancy of endometrial stem/progenitor cells in estrogen-depleted post-menopausal endometrium (128). As estrogen levels rise with thelarche and menarche, these potent stem/progenitor cells would commence proliferation and generation of clonal endometriotic tissue in the pelvic cavity and on the ovary. It is suggested that the overt bleeding observed in 5% of neonatal girls is indicative of a substantial “menses” with a greater degree of retrograde shedding and therefore greater risk of developing early onset endometriosis.

### Asherman's Syndrome

Asherman's syndrome (AS) is characterized by intrauterine adhesions/scarring and loss of a functional endometrium. Adhesions can be caused via surgical scraping/cleaning of the uterus or via uterine infection in a setting of low circulating estrogen e.g., post-partum and pregnancy termination. This trauma, to the endometrial basalis, causes loss of the germinal compartment for regenerating the endometrium. It has been proposed that trauma damages stem/progenitor populations and their surrounding stem cell niche in the basalis, preventing regeneration of the functionalis (40, 129, 130).

The use of endometrium-derived stem cells for treatment of Asherman's is still very much in its infancy. At the time of writing, few studies have investigated using menstrual fluid as a potential therapeutic option. Menstrual blood derived eMSC form spheroids that, when injected into the uteri of rats with induced AS, can improve fertility rates (131). In a small study of 7 human patients, autologous transfer of cultured MenSC resulted in an increase in endometrial thickness in 5 patients, four of which were able to undergo embryo transfer and two patients conceived successfully (132). Given menstrual fluid is a plentiful, easily available resource, research into autologous MenSC transfer would be worthy of further investigation.

## Conclusions

The endometrial stem cell field has advanced considerably since the first description of clonogenic cells in 2004. The recent identification of epithelial stem/progenitor markers has revealed a glandular epithelial hierarchy which likely supports re-epithelialization at menstruation as well as the growth of the functionalis during the proliferative phases. The remarkable regenerative capacity of endometrial stem cells shows promise for use in regenerative medicine, for endometrial disorders such as Asherman's but also for the treatment of infertility or miscarriage. The localization of eMSC and eEPC in menstrual and peritoneal fluid and ectopic lesions supports Leyendecker's theory that stem cells are involved in the pathogenesis of endometriosis, highlighting a potential target for future therapeutics. More importantly, the presence of both eMSC and eEPC in menstrual fluid has the potential to provide a new diagnostic tool for endometrial disorders. Advances in single cell sequencing will likely advance our understanding of the epithelial hierarchy and contributions of both eMSC and eEPC to the basalis and the functionalis.

## Author Contributions

FC was involved in the conception and design, acquisition of data, analysis and interpretation of the data, writing of the manuscript, editing and formatting the manuscript, and the drawing of the figures. CF provided acquisition, analysis and interpretation of data, writing of the manuscript, critical review of content, and drawing of figures. CG was involved in data acquisition, analysis and interpretation of data, writing of the manuscript, and critical review of content. All authors contributed to the article and approved the submitted version.

## Funding

This research was funded by an Australian National Health and Medical Research Council Investigator Fellowship to CG (1173882), an EndoFound Grant to CF and CG, and Victorian Government's Operational Infrastructure Support Program and the United States Department of Defense through the Congressionally Directed Medical Research Program under Award No. E01 W81XWH1910364 to CG.

## Author Disclaimer

Opinions, interpretations, conclusions, and recommendations are those of the author and are not necessarily endorsed by the Department of Defense.

## Conflict of Interest

The authors declare that the research was conducted in the absence of any commercial or financial relationships that could be construed as a potential conflict of interest.

## Publisher's Note

All claims expressed in this article are solely those of the authors and do not necessarily represent those of their affiliated organizations, or those of the publisher, the editors and the reviewers. Any product that may be evaluated in this article, or claim that may be made by its manufacturer, is not guaranteed or endorsed by the publisher.
